# Humanized Mice Exhibit Exacerbated Abscess Formation and Osteolysis During the Establishment of Implant-Associated *Staphylococcus aureus* Osteomyelitis

**DOI:** 10.3389/fimmu.2021.651515

**Published:** 2021-03-18

**Authors:** Gowrishankar Muthukrishnan, Alexandra Wallimann, Javier Rangel-Moreno, Karen L. de Mesy Bentley, Maria Hildebrand, Karen Mys, H. Mark Kenney, Eric T. Sumrall, John L. Daiss, Stephan Zeiter, R. Geoff Richards, Edward M. Schwarz, T. Fintan Moriarty

**Affiliations:** ^1^ Center for Musculoskeletal Research, Department of Orthopaedics and Rehabilitation, University of Rochester Medical Center, Rochester, NY, United States; ^2^ AO Research Institute Davos, Davos, Switzerland; ^3^ Swiss Institute of Allergy and Asthma Research (SIAF), University of Zurich, Davos, Switzerland; ^4^ Division of Allergy, Immunology and Rheumatology, Department of Medicine, University of Rochester Medical Center, Rochester, NY, United States; ^5^ Department of Pathology and Laboratory Medicine, University of Rochester Medical Center, Rochester, NY, United States

**Keywords:** humanized mice, *Staphylococcus aureus*, bone infection, osteolysis, staphylococcal abscess communities, T cells

## Abstract

*Staphylococcus aureus* is the predominant pathogen causing osteomyelitis. Unfortunately, no immunotherapy exists to treat these very challenging and costly infections despite decades of research, and numerous vaccine failures in clinical trials. This lack of success can partially be attributed to an overreliance on murine models where the immune correlates of protection often diverge from that of humans. Moreover, *S. aureus* secretes numerous immunotoxins with unique tropism to human leukocytes, which compromises the targeting of immune cells in murine models. To study the response of human immune cells during chronic *S. aureus* bone infections, we engrafted non-obese diabetic (NOD)–*scid* IL2Rγ^null^ (NSG) mice with human hematopoietic stem cells (huNSG) and analyzed protection in an established model of implant-associated osteomyelitis. The results showed that huNSG mice have increases in weight loss, osteolysis, bacterial dissemination to internal organs, and numbers of Staphylococcal abscess communities (SACs), during the establishment of implant-associated MRSA osteomyelitis compared to NSG controls (*p* < 0.05). Flow cytometry and immunohistochemistry demonstrated greater human T cell numbers in infected versus uninfected huNSG mice (*p* < 0.05), and that T-bet^+^ human T cells clustered around the SACs, suggesting *S. aureus*-mediated activation and proliferation of human T cells in the infected bone. Collectively, these proof-of-concept studies underscore the utility of huNSG mice for studying an aggressive form of *S. aureus* osteomyelitis, which is more akin to that seen in humans. We have also established an experimental system to investigate the contribution of specific human T cells in controlling *S. aureus* infection and dissemination.

## Introduction

Bone infections, a debilitating complication of total joint replacement (TJR) arthroplasties and fracture fixation, have dramatically increased over the past decade in the United States alone (1–3). *Staphylococcus aureus*, a significant human pathogen, remains the leading cause of bone infections in TJR surgeries, causing 30-42% of fracture-related infections (FRI), and 10,000-20,000 peri-prosthetic joint infections (PJI) in patients each year in the US (4–7). Methicillin-resistant *S. aureus* (MRSA) and newly emerging strains with pan-resistance significantly complicate treatment leading to adverse clinical outcomes such as amputation and septic death (8, 9).

There is an urgent need to control these deep bone infections utilizing non-antibiotic interventions. Unfortunately, no preventative *S. aureus* immunotherapies exist, despite almost 20 years of research to identify conceptually promising vaccine targets and significant money spent on clinical trials (10–12). Poor antigen selection and the ability of *S. aureus* to evade the human immune system might contribute to the failure of vaccines. Alternatively, the lack of relevant models that recapitulate human immune responses could explain the failure of these trials.

Murine models have greatly facilitated our understanding of *S. aureus* pathogenesis and identified critical virulence factors such as staphylococcal protein A, iron-scavenging proteins, fibrinogen binding proteins, penicillin-binding proteins, hemolysins, autolysins, etc. (13–23). However, the knowledge acquired using these murine models does not necessarily translate into these targets becoming useful vaccine candidates in humans. A prominent case in point is the murine preclinical data of an immunogenic vaccine candidate from iron-scavenging protein IsdB (IsdB-V710) that demonstrated reduced infection lethality, and protection against bacteremia in mice (24–27). Unfortunately, a large phase IIb/III vaccination clinical trial based on these preclinical studies involving ~8,000 patients failed to provide any protection and elevated the risk of adverse outcomes, including death, among patients who encountered post-immunization *S. aureus* infections (28). Therefore, we are in dire need of small animal models that can better mimic the human immune system. Moreover, *S. aureus* is a significant human pathogen with several virulence proteins and bicomponent toxins with high degrees of tropism to receptors expressed on human leukocytes (29, 30). Due to these human-specific toxins, it is possible that this pathogen does not necessarily exhibit their typical phenotype in murine *S. aureus* infections.

Non-obese diabetic (NOD)–*scid* IL2Rγ^null^ (NSG) mice, reconstituted with human CD34+ hematopoietic immune system (huNSG), have emerged as a powerful model system to investigate human disease (31–33). These mice evoke a human immune response to infection and have been utilized to study bacterial and viral pathogens such as *Salmonella*, *Leishmania*, HIV, and EBV (34–39). The use of humanized mice to study *S. aureus* infections remains relatively limited (40–42), and until now, no studies have described *S. aureus* pathogenesis during osteomyelitis in humanized mice. To this end, we developed a transtibial implant-associated *S. aureus* osteomyelitis model in humanized NSG mice and examined if *S. aureus* induces a human immune response in these mice during bone infection. Additionally, we also assessed infection severity, the extent of bone osteolysis, and Staphylococcal abscess communities (SAC) formation during the establishment of implant-associated MRSA osteomyelitis.

## Materials and Methods

### Ethics Statement

Animal studies were performed according to protocols approved by the ethical committee of the canton of Grisons in Switzerland. Animal surgical procedures were performed according to Swiss animal protection law and regulations in an Association for Assessment and Accreditation of Laboratory Animal Care (AAALAC) International approved facility.

### Murine Implant-Associated Osteomyelitis Model

Female C57BL/6J mice (stock 000664), NSG (NOD.Cg-Prkdc^scid^ Il2rg^tm1Wjl^/SzJ, stock 005557) mice were purchased from the Jackson Laboratories (Bar Harbor, ME, USA), housed five per cage in two-way housing on a 12-h light/dark cycle, and fed a maintenance diet and water *ad libitum*. Humanized NSG (huNSG) mice were generated by Jackson Labs by engrafting NSG mice with CD34+ human hematopoietic cells from three different donors using protocols described previously (31, 32). Briefly, 3-week old NSG mice were subjected to total body irradiation (100 cGy) and injected intravenously with lineage negative human CD34^+^ hematopoietic stem cells (2 x 10^5^ cells/mice) isolated of cord blood. At 12 weeks post engraftment, mice were subjected to submandibular bleeding to isolate peripheral lymphocytes and human immune cell reconstitution was assessed in huNSG mice by flow cytometry (markers: anti-human CD45 - overall reconstitution, anti-human CD3 – T cells, anti-human CD20 – B cells, anti-human CD33 – myeloid cells). **Supplemental Table 1** describes the percentage of human CD45+ cells, human B cells, T cells, and myeloid cells engrafted in huNSG mice generated from all different donors. Transtibial implant-associated osteomyelitis with MRSA was performed on skeletally mature 20–24-week-old huNSG mice, and age-matched C57BL/J6 and NSG mice utilizing our well-validated protocols described previously (22, 43, 44). Briefly, mice were anesthetized with Sevoflurane in a Plexiglass box (ca. 7% in O2, flow rate 0.6-1 L/min), maintained with Sevoflurane through a face mask (ca. 2-3% in O2, flow rate 0.6-1 L/min). Peri- and postoperative analgesia consisted of Tramal, which was added to the drinking water 24h prior to surgery (25mg/L) and maintained for two days after surgery to minimize skin wounds from injections and at the same time provide adequate analgesia. Before surgery, a flat stainless-steel surgical wire (cross-section, 0.2 mm by 0.5 mm) 4 mm long (MicroDyne Technologies, Plainville, CT, USA) bent at 1mm to form an L-shape was steam sterilized and inoculated with clinical *S. aureus* USA300 LAC strain grown overnight. After anesthesia induction, the right leg was clipped, and the skin was aseptically prepared with chlorhexidine scrub (Hibiscrub, 4% Chlorhexidine Digluconate) and 70% ethanol. The implant localization was identified (2 to 3 mm under the tibial plateau in the proximal tibia) using the proximal patella as an anatomical landmark and the jaws of the Mayo-Hegar needle driver as the measure. A hole was pre-drilled in the proximal tibia using a percutaneous approach from the medial to lateral cortex using a 26-gauge needle. Subsequently, a *S. aureus* infected pin (5.0 x 10^5^ colony forming units (CFU)/mL) was surgically implanted in the pre-drilled hole from the medial to the lateral cortex. Osteotomy and implant position were confirmed radiographically in the lateral plane immediately after surgery. At 14 days post-infection, mice were euthanized, and the infected leg containing the transtibial implant was excised out for either CFU quantitation or high-resolution micro-computed tomography (μCT) imaging, followed by histology and transmission electron microscopy (TEM). Additionally, internal organs liver, spleen, kidneys, and heart were harvested sterilely for CFU enumeration. Further, all mice were subjected to submandibular bleeding on days 0, 7, and 14 post-infection to collect serum for assessing anti-*S. aureus* antibodies. Murine infection studies were performed four independent times and the results shown are pooled data from these experiments.

### Bacteriology

Tibia, tibial implant, and the soft tissue abscesses surrounding the tibia were removed, weighed, and placed in 1mL of room temperature sterile PBS. The implant was sonicated for 2 min to dislodge attached bacteria, and organ tissues were homogenized (Omni TH, tissue homogenizer TH-02/TH21649, Kennesaw, GA, USA) in 1mL of PBS. Implant sonicate fluid and tissue homogenates were serially diluted, plated on blood agar (BA) plates, and incubated overnight at 37°C. To confirm *S. aureus* on the plates, random colonies from each plate/organ/tissue were picked, and StaphLatex agglutination test (Thermo Fisher Scientific, Waltham, MA, USA) was performed. Bacterial colonies were enumerated, and the generated CFU data were presented as CFUs per gram of tissue.

### Micro-Computed Tomography (μCT)

The tibia was dissected from mice post-euthanasia and fixed for 72 hours in 4% neutral buffered formalin. Subsequently, specimens were rinsed in PBS, deionized water, and prepared for μCT scans. High-resolution μCT scans of the mice tibia receiving MRSA-contaminated or sterile pin were imaged ex vivo at 10.5 μm voxel size with the VivaCT40 (Scanco Medical AG, Switzerland), using 100 ms integration time, energy of 70 kV, and intensity of 114 μA. Post-processing and analyses of the resultant DICOM files generated from VivaCT40 were performed on Amira software (FEI Visualization Sciences Group; Burlington, MA, USA). Medial and Lateral hole volume quantification was performed by manual segmentation of the void area followed by a point trap triangulation in Amira. Reactive bone volume was also computed using methods described previously by Mys et al. (45). Briefly, the bone was segmented using adaptive thresholding techniques and masks described previously (45). Then, the thickness of all bone structures was calculated in IPL software (Scanco Medical AG, Switzerland), and all the bone structures thicker than 6 voxels (63.0μm) were assigned to be cortex. The reactive bone was calculated by subtracting the quantified outer mask from the cortex. Thresholding was set at 10 voxels to clean the reactive bone masks. The reactive bone volume calculations were performed only on the distal side of the pin to minimize the influence of the pin’s position on the results.

### Histology

Following μCT, each mouse tibia was rinsed with ddH2O and decalcified in 14% EDTA tetrasodium solution for 7 days, with radiographical monitoring of the decalcification progress. Following decalcification, samples were paraffin-embedded, cut into 5 μm transverse sections, and mounted on glass slides for histological staining. Slides were deparaffinized and stained with Hematoxylin & Eosin (H&E) and Brown and Brenn (Gram) staining as described previously (43, 46). Digital images of the stained slides were created using VS120 Virtual Slide Microscope (Olympus, Waltham, MA, USA). Numbers SACs were manually enumerated and averaged across two or more histologic sections at least 50 μm apart from 6-7 mice in each experimental group. Quantitative analysis of SAC area within the tibias of C57BL/6J WT, NSG, and huNSG animals was performed on Brown and Brenn (Gram) stained slides using Visiopharm (v.2019.07; Hoersholm, Denmark) colorimetric histomorphometry utilizing a custom Analysis Protocol Package (APP). Manual regions-of-interest (ROIs) were drawn around the tibia and SACs within the tibia on each image prior to batch processing for automated quantification of SAC area normalized to tibial area between the groups.

### Multicolor Immunofluorescence

*Primary antibodies:* The following antibodies were utilized for immunostaining: Goat anti-CD3ε (Clone M-20, Santa Cruz Biotechnology, Dallas, TX, USA, RRID : AB_631128), goat anti-proliferating cell nuclear antigen (Clone C-20, Santa Cruz Biotechnology) at 1:100 dilution, Rabbit anti-human CD20 at 1:50 dilution (LS-B2605-125, LifeSpan Biosciences, Seattle, WA, USA, RRID : AB_10439766), biotin rat anti-mouse Ly6G at 1:50 dilution (Clone 1A8, BioLegend, Austin, TX, USA, RRID : AB_1186108), rabbit anti-Tbet at 1:50 dilution (clone H-210, Santa Cruz Biotechnology), and monoclonal mouse anti-human RORγT at 1:50 dilution (clone 6F3.1, EMDMilipore, Burlington, MA, USA, RRID : AB_11205416). *Secondary antibodies:* All secondary antibodies were used at 1: 200 dilution. These include Alexa Fluor 568 donkey anti-goat IgG (A-11057, Thermo Fisher Scientific, RRID : AB_2534104), Alexa fluor 488 donkey anti-rabbit IgG (711-546-152, Thermo Fisher Scientific, RRID : AB_2340619), Alexa fluor 647 donkey anti-rat IgG (712-606-153, Jackson ImmunoResearch Laboratories, West Grove, PA, USA, RRID : AB2340865), Alexa fluor 647 donkey anti-mouse IgG (715-606-150, Jackson ImmunoResearch Laboratories, RRID : AB2340865), and Alexa Fluor 680 Streptavidin at 1:200 dilution (S32358, Thermo Fisher Scientific).

The 5 μm formalin-fixed paraffin sections were incubated at 60°C overnight for deparaffinization. Tissue sections were quickly transferred to xylene and gradually hydrated by transferring slides to absolute alcohol, 96% alcohol, 70% alcohol, and then water. Slides were immersed in an antigen retrieval solution, boiled for 30 minutes, and cooled down for 10 minutes at room temperature (RT). Slides were rinsed several times in water and transferred to PBS. Non-specific binding was blocked with 5% normal donkey serum in PBS containing 0.1% Tween 20, 0.1% Triton-X-100 for 30 minutes, at RT in a humid chamber. Primary antibodies were added to slides and incubated in a humid chamber at RT, ON. Slides were quickly washed in PBS, and fluorescently labeled secondary antibodies were incubated for 2 hours at RT overnight in a humid chamber. Finally, slides were rinsed for 1 hour in PBS and mounted with Vectashield antifade mounting media with DAPI (H-1200, Vector Laboratories, Burlingame, CA, USA). Pictures were taken with a Zeiss Axioplan 2 microscope and recorded with a Hamamatsu camera.

### Transmission Electron Microscopy (TEM)

Brown and Brenn staining was performed to identify SAC presence within the intramedullary canal of MRSA-infected huNSG mice. Once a SAC was identified, the paraffin block was oriented to match the 5 μm section of the Brown and Brenn slide, in order to excise the precise area from the paraffin block. Once the right area was excised, the block was deparaffinized, post-fixed sequentially in 2.5% glutaraldehyde (24 hours) and 1.0% osmium tetroxide (90 minutes), dehydrated in a graded series of ethanol to 100%, transitioned into propylene oxide, infiltrated with EPON/Araldite epoxy resin and finally embedded block face down into a BEEM capsule lid for 48 hours at 60°C. The block was sectioned at one micron and stained with Toluidine blue to confirm the SAC location, then thin sectioned at 70 nm using a diamond knife and an ultramicrotome. The thin sections were mounted onto formvar carbon coated nickel slot grids, then examined using a Hitachi 7650 transmission electron microscope, and images were captured using Gatan Erlangshen 11-megapixel digital camera and DigitalMicrograph software.

### Flow Cytometry

Immunophenotyping of spleen from huNSG mice was performed according to protocols described previously (47). Briefly, single-cell suspension of splenocytes were prepared, and 0.5 X 10^6^ cells/mice were initially stained with fixable viability dye eFluor™ 780 (eBioscience™, Thermo Fisher Scientific) for 30 minutes at 4° C to exclude dead cells from the analysis. Following washing, the following fluorochrome-conjugated anti-human antibodies were used for phenotyping huNSG splenocytes: BV510 CD45 (clone 2D1), PerCP CD3 (clone UCHT1), PE-Dazzle 594 CD8a (clone HIT8a), FITC CD4 (clone OKT4), PE-Cy5 CD19 (clone SJ25C1), and PE CD56 (clone HCD56). Single channel compensation controls for these antibodies were created using human polymorphonuclear cells (PMBCs). All antibodies were purchased either from BioLegend or BD Biosciences (San Jose, CA, USA). After staining, the cells were fixed with 2% formaldehyde/PBS prior to running on a BD FACSAria™ III multicolor flow cytometer (BD Biosciences). Flow data were analyzed using FlowJo version 10.6 (BD Biosciences), and the gating strategies are outlined in **Supplemental Figure S1**.

### Serum Cytokine and Anti-Human Cytokine and Antibody Measurements

HuNSG mice infected with either a sterile (Sham) or *S. aureus* contaminated tibial implant were bled submandibularly to collect serum samples PreOP, at day 7 and day 14 post infection as allowed under the Swiss animal protection regulations, and the protocols approved by the ethical committee of the canton of Grisons in Switzerland. Serum cytokine analyses was performed using a 25-plex MILLIPLEX^®^ xMAP Human cytokine Magnetic Bead Panel for the following cytokines according to manufacturer’s instructions: GM-CSF, IFN-γ, IL-1β, IL-2, IL-4, IL-5, IL-6, IL-9, IL-10, IL-12 (p70), IL-13, IL-15, IL-17A, IL-17F, IL-17E/IL-25, IL-21, IL-22, IL-23, IL-27, IL-28A, IL-31, IL-33, MIP-3α/CCL20, TNF-α, and TNF-β. Only 7 out of the 25 analytes expressed at detectible levels: IFN-γ, CCL20, IL-13, IL-9, IL-21, IL-17E/IL-25, and TNF-α. The manufacturer defined assay sensitivity or Lower Limits of Detection for these cytokines are as follows: IFN-γ = 2.4 pg/mL, CCL20 = 3.4 pg/mL, IL-13 = 3.5 pg/mL, IL-9 = 8.7 pg/mL, IL-21 = 3.3 pg/mL, IL-17E/IL-25 = 0.186 pg/mL, and TNF-α = 1.7 pg/mL. Additionally, using our previously validated Luminex bead-based immunoassay (48–50), anti-*S. aureus* human antibody responses in serum were assessed 14 days post-infection in huNSG mice with the following *S. aureus* antigens: iron-regulated surface determinant proteins (IsdA, IsdB, and IsdH), the staphylococcal complement inhibitor (SCIN), the chemotaxis inhibitory protein from *S. aureus* (CHIPS), α-hemolysin (Hla), autolysin (Atl) functional domains amidase (Amd) and glucosaminidase (Gmd), and Leukocidin LukSF-PV/PVL (LukS-PV, LukF-PV).

### Statistical Analyses

Unpaired student’s t-test was used for statistical comparison of the flow cytometry data. Two-way ANOVA with Sidak’s post-hoc tests was performed to compare body weight change over time. One-way ANOVA analyses with Tukey’s post-hoc tests were utilized for comparing osteolysis area, number of SACs, SAC area, log-transformed CFUs, and the number of immune cells revealed by immunostaining. All analyses were conducted using GraphPad Prism (version 9.0), and *p* < 0.05 was considered significant.

## Results

### Humanized NSG Mice Elicit Human T Cell Responses During the Establishment of *S. aureus* Osteomyelitis

**Figure 1:**
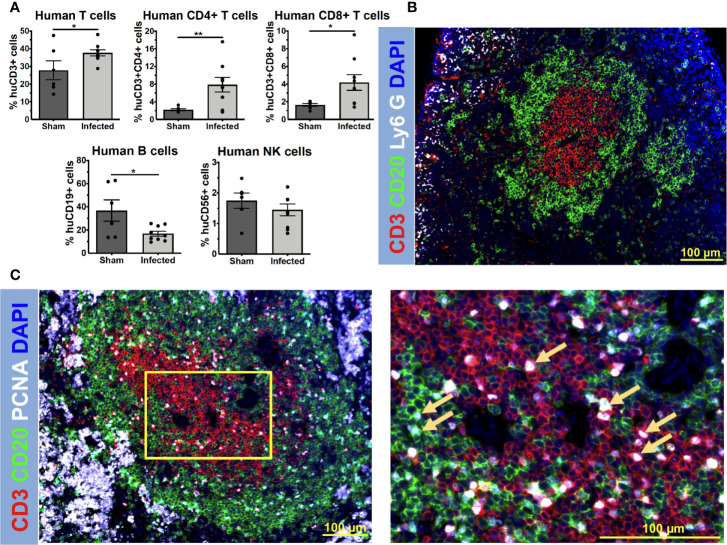
*S. aureus* elicits a human immune response in humanized NSG mice. HuNSG mice received a sterile (Sham) or MRSA contaminated transtibial implant, and 14 days post infection, mice were euthanized, and spleens were harvested for analyses. Single-cell suspensions of splenocytes were prepared and subjected to immunophenotyping analyses by flow cytometry with anti-human mAbs to assess human T cells (CD3+), T helper cells (CD3+CD4+), cytotoxic T cells (CD3+CD8+), B cells (CD19+), and NK cells (CD56+). The sequential gating strategy is depicted in **Supplemental Figure S1**. **(A)** The percentage of each lymphocyte subset of the live/human CD45+ cells analyzed is presented for each mouse with the mean +/- SD for the group (n = 15, *p < 0.05, **p < 0.01, t-test). **(B)** Paraffin-embedded 5 μm spleen sections from infected humanized NSG mice were stained to visualize the spatial distribution of human CD3^+^ T cells (red), human CD20^+^ B cells (green), and murine Ly6G^+^ neutrophils (white). Representative 3x3 200x mosaic immunofluorescent images are shown highlighting the compartmentalization of interacting human T and B cells in spleens of infected huNSG mice. **(C)** Adjacent 5 μm spleen sections were also stained for examining cell proliferation in response to *S. aureus* infections using proliferating cell nuclear antigen (PCNA) (white). Yellow squares show higher magnification images of proliferating CD3^+^ T cells (red), CD20^+^ B cells (green) (PCNA+ cells, yellow arrows), in the sections of the 3x3 mosaic immunofluorescent micrographs.

Because NSG mice allow the engraftment of human immune cells, we hypothesized that MRSA infection would elicit a human immune response in huNSG mice (**Supplemental Table S1**). To test this, huNSG mice received a sterile (Sham) or MRSA contaminated tibial implant, and the spleens were harvested for analyses on day 14 post-op. Immunophenotyping by flow cytometry revealed that *S. aureus* infection induced significant upregulation of human CD3+ T cells (*p* = 0.029) and its subsets CD4+ T helper cells (*p* = 0.007), CD8+ cytotoxic T cells (*p* = 0.019) in huNSG compared to the control group (**Figure 1A**). No such induction of human CD19+ B cells or CD56+ natural killer (NK) cells were observed in infected huNSG. Additionally, immunofluorescent histochemistry revealed distinctive B and T cell areas in the spleen of infected huNSG mice (**Figure 1B**). Additionally, immunostaining with human cell proliferation marker PCNA revealed expanding human T and B cells in huNSG mice in response to *S. aureus* (**Figure 1C** **)**. However, anti-*S. aureus* human antibody responses in huNSG serum using our custom Luminex assay were undetectable 14 days post infection (data not shown), and serum cytokine levels analyzed over time revealed modest induction of human cytokines including IFN-γ, TNF-α, and IL-13 (**Supplemental Figure S2**). Nonetheless, our results indicate that *S. aureus* infection induces a human immune response in the spleen of huNSG mice.

### Humanized NSG Mice Exhibit Exacerbated Susceptibility to *S. aureus* Osteomyelitis

**Figure 2:**
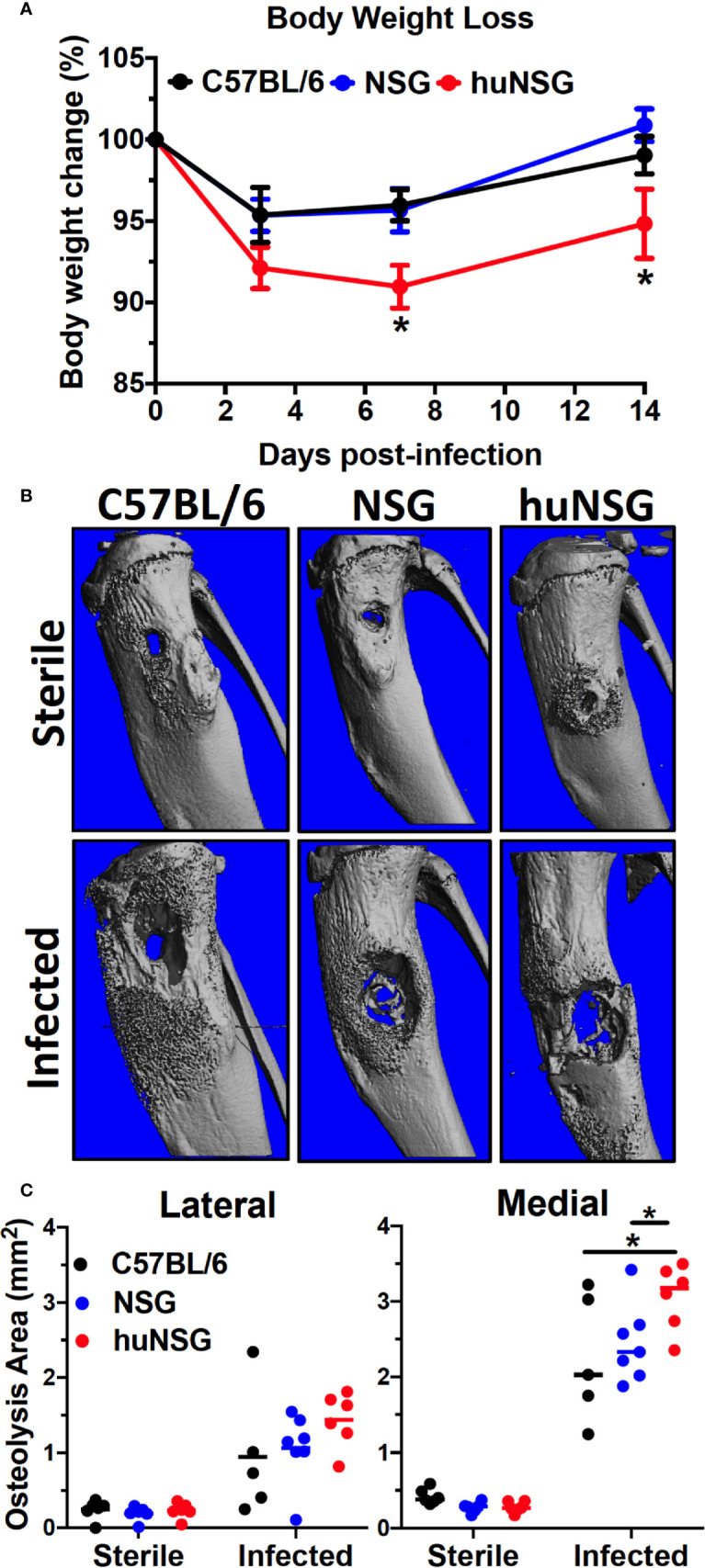
Humanized mice exhibit increased body weight loss and osteolysis during *S. aureus* implant-associated osteomyelitis. **(A)** HuNSG mice and age-matched C57BL/6 WT, NSG controls underwent transtibial implantation of MRSA (USA300 LAC) contaminated stainless steel wire, and total body weight was assessed over the 2-week infection period. The % of baseline body weight on days 0, 3, 7 and 14 is presented for each group with the mean +/- SD (n = 14-17, *p < 0.05, two-way ANOVA). **(B)** Tibiae implanted with sterile and MRSA contaminated wires were harvested on day 14 post-op, processed for μCT, and representative 3D renderings are shown to illustrate the levels of reactive bone formation and osteolysis around the implants. Note the extensive osteolysis in the infected huNSG tibia. **(C)** The osteolysis area on the lateral and medial sides of the tibiae were quantified, and the data for each is presented with the mean +/- SD for the group (n = 6, *p < 0 .05, one-way ANOVA). Note that osteolysis is greater on the medial side in this model due to the directionality of wire implantation from the medial to the lateral side.

Given the potential negative impact of *S. aureus* immunotoxins on human immune cells, we hypothesized that the huNSG mice would develop a more severe MRSA infection due to the presence and induction of human immune system. To test this, we examined implant-associated osteomyelitis in huNSG mice and its age-matched NSG, C57BL/6J WT counterparts. In general, huNSG mice appeared sicker, failed to recover their body weight after implant surgery, and exhibited significantly increased weight loss throughout the 14-day study period (**Figure 2A**, *p* < 0.05). High-resolution μCT analyses of the tibiae revealed that *S. aureus*-infected huNSG mice displayed significantly greater peri-implant osteolysis at the insertion site compared to age-matched NSG and C57BL/6J WT controls (**Figures 2B, C**, *p* < 0.05). Interestingly, no differences in reactive bone volume were observed between these groups, suggesting that the human engrafted cells do not affect osteoblast activity (**Supplemental Figure S3**). No difference in osteolysis was observed in animals that underwent sterile-implant surgery, suggesting that the observed bone phenotype is due to *S. aureus* infection.

**Figure 3:**
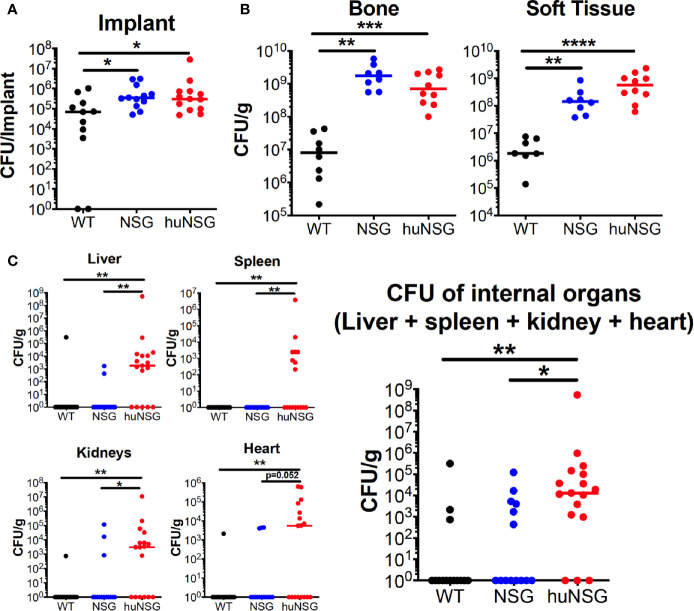
Humanized mice exhibit increased bacterial load at the surgical site and sepsis during *S. aureus* implant-associated osteomyelitis. WT, NSG, and huNSG mice received MRSA infected trans-tibial implants and were euthanized on day 14 post-op to quantify CFUs from the **(A)** implant, **(B)** tibia and adjacent soft tissue, and **(C)** internal organs. The data are presented for each mouse with mean +/- SD for the Group (n = 14-17, *p < 0.05, **p < 0.01, ***p < 0.001, one-way ANOVA).

To assess effects of human engrafted cells on bacterial load, ex vivo CFU quantification was performed on the implants, which revealed higher bacterial loads in huNSG (13-fold, *p* = 0.012) and NSG (4.2-fold, *p* = 0.025) mice compared to C57BL/6J WT (**Figure 3A**). Similarly, an 86.2- to 215.2-fold higher CFU on the tibia (*p* < 0.01) and a 79.7- to 310.9-fold higher CFU load on infected soft tissue (*p* < 0.01) surrounding the bone were observed in huNSG and NSG mice (**Figure 3B**). Interestingly, significantly increased MRSA dissemination from the implant to internal organs (kidney, liver, heart, and spleen) was observed in huNSG compared to control groups (**Figure 3C**). 14/17 huNSG mice were *S. aureus* culture-positive from at least one organ, while only 6/14 and 3/14 mice in the NSG and WT groups were culture-positive in at least one organ (**Figure 3C**). Remarkably, some huNSG mice were highly septic due to MRSA bone infection, while some huNSG mice showed no dissemination (**Figure 3C**).

**Figure 4:**
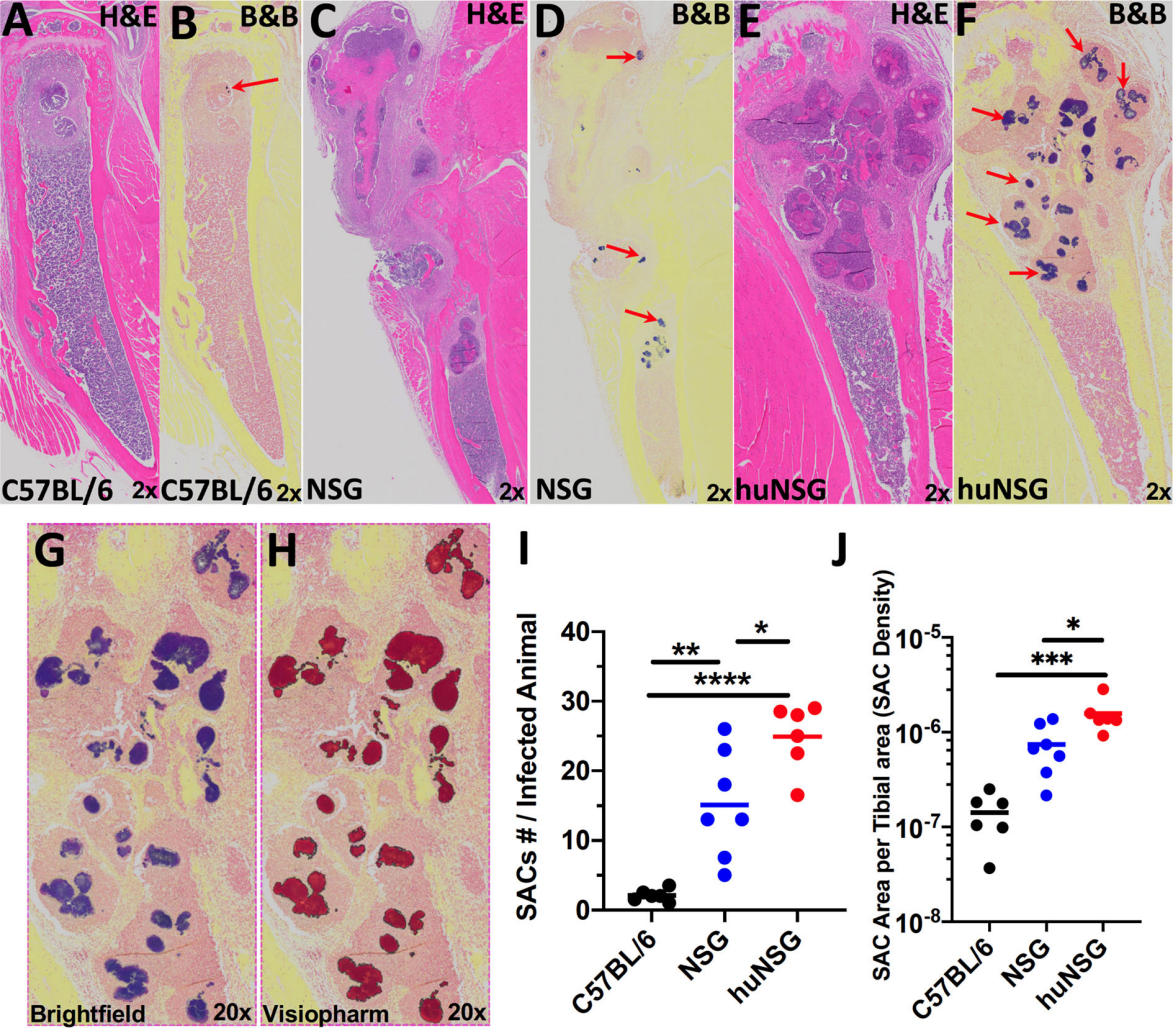
Humanized mice exhibit increased Staphylococcal abscess community (SAC) formation. WT, NSG, and huNSG mice received MRSA infected transtibial implants, and the tibiae were harvested on day 14 post-op for histology and assessment of SACs. Representative micrographs of H&E **(A, C, E)** and Brown & Brenn Gram **(B, D, F)** stained sections are presented to illustrate the abscesses and Gram-positive bacteria (red arrows). **(G, H)** Digital scans of the histology were processed by Visiopharm software, which recognized the Gram-stained bacteria, and scored the positive pixels (purple to red color conversion) for automated histomorphometry of SAC numbers per tibia **(I)** and SAC area per tibia **(J)**. The data are presented for each mouse with the mean for each Group (n = 6-7, *p < 0.05, **p < 0.01, ***p < 0.001 one-way ANOVA).

**Figure 5:**
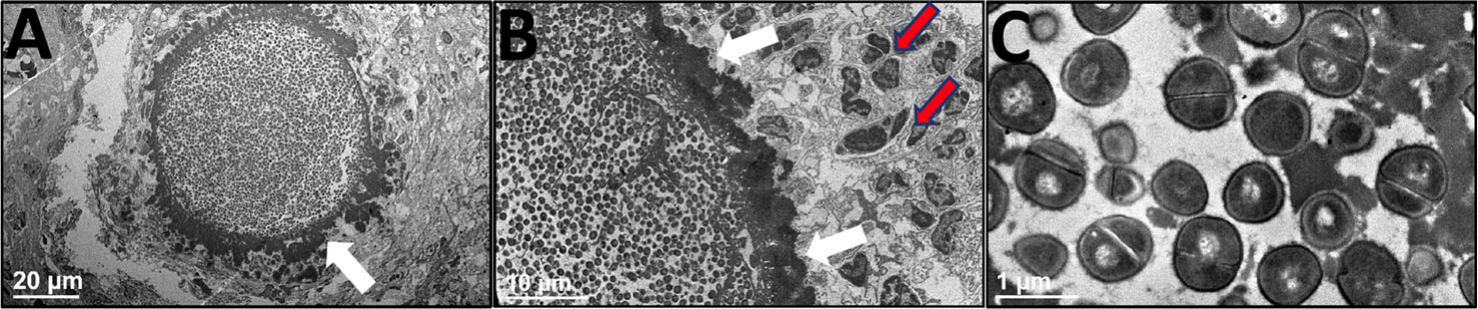
Ultrastructural assessment of Staphylococcal abscess communities (SACs) in humanized mice. HuNSG mice were subjected to MRSA transtibial infection and tibiae were harvested 14 days post-op, formalin-fixed, and decalcified for TEM processing. These paraffin-embedded tibiae samples were reprocessed into epoxy resin for transmission electron microscopy. A representative SAC in the bone marrow cavity is shown in **(A)** x1200 and **(B)** x3000 to illustrate the bacteria within the electron dense pseudocapsule (white arrows), and adjacent immune cells (red arrows) that are unable to penetrate the SAC. **(C)** High magnification of *S. aureus* bacteria within the SAC (x 30,000).

Next, histopathology of the infected tibia was performed to further assess the extent of bone osteolysis in huNSG mice. H&E staining of the infected tibia confirmed the extensive osteolysis revealed in huNSG mice (**Figure 4E**) compared to C57BL/6 WT (**Figure 4A**) and NSG (**Figure 4C**) controls. In addition, Brown and Brenn staining of the infected tibia revealed extensive Staphylococcal abscess communities (SAC) formation in huNSG mice (**Figure 4F**) compared to control groups (**Figures 4B, D** **)**. The number of SACs formed per infected tibia was significantly higher in huNSG than in control groups (**Figure 4I**, *p* < 0.05). Histomorphometry quantification revealed a marked increase in the SAC area in huNSG, suggesting heightened severity of MRSA bone infection in these animals (**Figures 4G–J**, *p* < 0.05). TEM interrogation of mature SACs in huNSG mice confirmed the formation of a fibrin-like pseudocapsule (51, 52), which sequesters and protects *S. aureus* from host immune cells (**Figure 5**).

### Induction of Human T Cell Response in huNSG Tibiae Due to *S. aureus* Osteomyelitis

**Figure 6:**
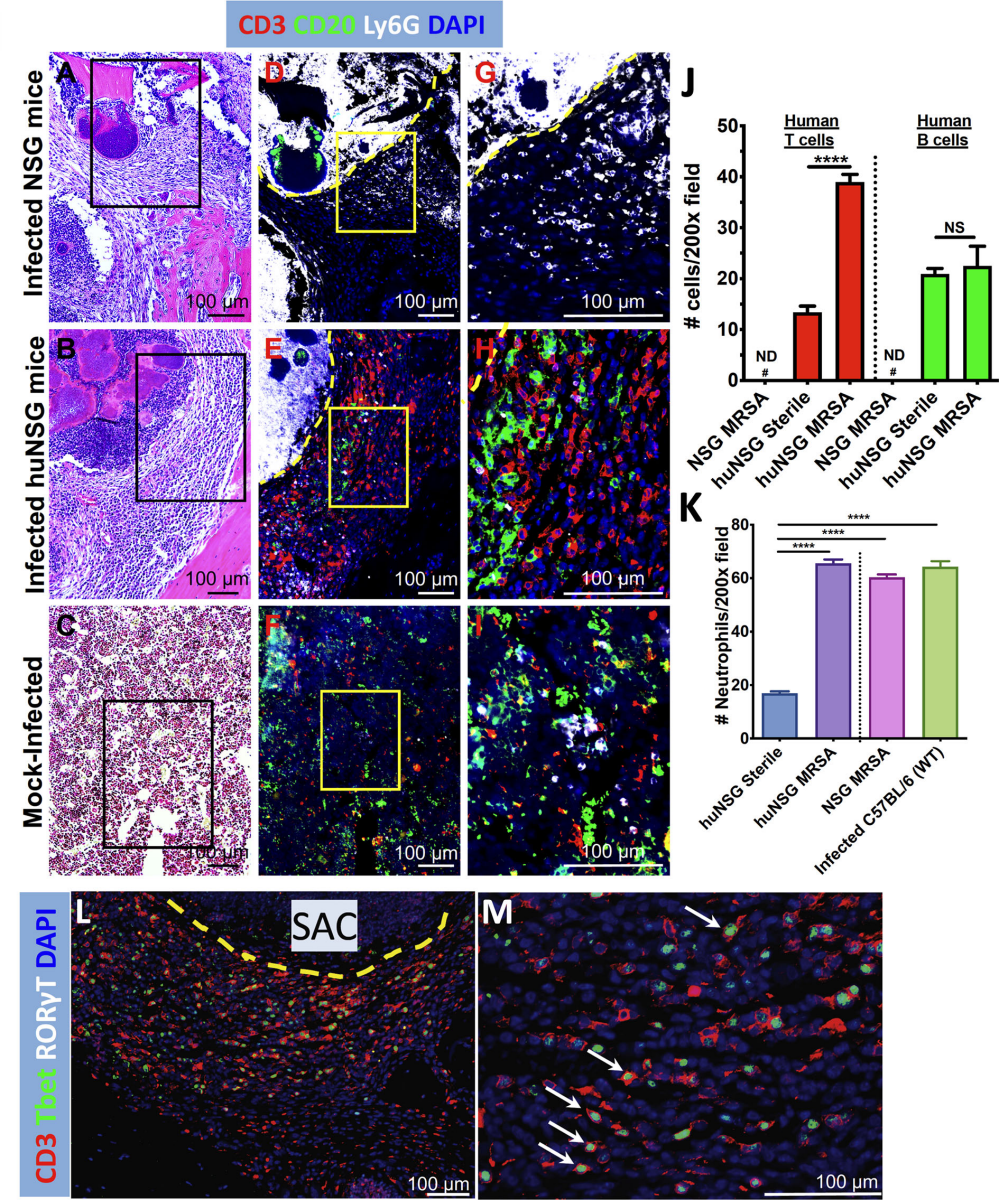
Evidence of human T cell immune responses against *S. aureus* in huNSG mice with implant-associated osteomyelitis. The histology sections (n = 3-5 per Group) described in **Figure 4** were stained with fluorescently labeled antibodies specific for anti-mouse Ly6G, anti-human CD3, anti-human CD20, anti-human Tbet, and anti-human RORγT. Light microscopy of the H&E stained sections **(A–C)**, and fluorescent microscopy of adjacent 5 μm sections **(D–I)** were performed on the SACs in tibiae from infected NSG, infected huNSG mice, and sham-control huNSG mice. Black squares in H&E images show the area depicted in 3x3 mosaic immunofluorescent micrograph. Yellow squares show higher magnification images of the CD3^+^ T cells (red), CD20^+^ B cells (green), and Ly6G^+^ neutrophils (white), in the sections of the 3x3 mosaic immunofluorescent micrographs. The dotted yellow line separates the SAC border from the rest of the bone marrow. Note that mouse Ly6G^+^ neutrophils accumulated inside and in close proximity to SACs, and the absence of human lymphocytes in infected NSG mice **(D, G)**. In contrast, large numbers of human T and B cells accumulate around the SACs in the infected huNSG mice **(E, H)**, while human lymphocytes are scant in uninfected huNSG mice **(F, I)**. Histomorphometry was performed on 5 randomly chosen fields at 200X magnification in each condition **(J, K)**, and aggregated data is presented as the mean+/- SEM for each Group (n = 3-5 mice, ND, not detected, NS, not significant, ****p < 0.0001, one-way ANOVA). **(L, M)** Evidence of Type 1 human T cell induction (CD3+T-bet+, white arrows) adjacent to the SACs.

We next investigated the repertoire and spatial distribution of human T and B cells proximal to SACs *via* multicolor immunofluorescent histochemistry (**Figure 6**). The tibia sections from infected huNSG mice revealed significant induction and trafficking of human T cells clustered adjacent to SACs (**Figures 6B, E, H, J**, *p* < 0.0001). Human B cells were observed in sham treated huNSG mice, but only small amounts of these cells were induced and trafficked in response to *S. aureus* infections (**Figure 6J**). Expectedly, no human B or T cells were identified in nonengrafted NSG control mice (**Figures 6A, D, G** **)**, though *S. aureus* induced production of mouse Ly6G^+^ neutrophils in the infected tibia of huNSG and NSG animals (**Figure 6K**, *p* < 0.05). Interestingly, the levels of murine Ly6G^+^ neutrophils in these animals were similar to the levels observed in C57BL/6 WT animals in response to *S. aureus* (**Figure 6K** **)**. Besides, the presence of mouse Ly6G^+^ neutrophils in huNSG suggests recovery of innate cells post γ-irradiation-induced myeloablation in NSG mice before HSC engraftment. Subsequent immunofluorescent staining of infected huNSG tibiae revealed CD3+ T-bet+ Type 1 human T cells adjacent to the SACs (**Figures 6L, M** **)**. In addition, examination of huNSG tibia sections using proliferating cell nuclear antigen (PCNA) revealed that both human T and B cells are proliferating near the SACs and that the percentage of proliferating human T cells (CD3+PCNA+ cells) is significantly higher than that of B cells (CD20+PCNA+ cells) (**Supplemental Figure S4**). Collectively, these results suggest *S. aureus*-mediated activation and proliferation of type 1 human T cells.

## Discussion

Development of effective immunotherapies against *S. aureus* remains among the greatest priorities in orthopedics as bone infections caused by this pathogen continue to be a significant public health problem (1). The failure of several anti-*S. aureus* vaccine trials can be attributed to overreliance on preclinical murine studies, where *S. aureus* does not entirely display their typical phenotype (10, 53). Humanized mice have emerged as an attractive small animal model to investigate human disease (54). In the current study, we assessed its utility to study *S. aureus* pathogenesis during implant-associated osteomyelitis. In this proof-of-concept study involving *S. aureus* transtibial implant-associated osteomyelitis in huNSG mice, we observed that these mice displayed increased susceptibility to *S. aureus* as evidenced by increased weight loss and extensive peri-implant osteolysis compared to C57BL/6 mice. Others have shown that huNSG mice also display increased susceptibility to *S. aureus* infection in peritoneum, skin, and lung infection models (40–42), though these studies were acute infection studies unlike the one described here. Importantly, the authors noted that huNSG mice required 10-100-fold fewer bacteria to have analogous pathology in the non-humanized mice (41). In our model, it is conceivable that the more severe infection phenotype in huNSG mice could be the result of higher bacterial inoculum that we routinely use for achieving reproducible implant-associated osteomyelitis in C57BL/6 mice (22, 23, 43, 44, 55). Nonetheless, this critical finding needs to be carefully examined in our humanized mouse model of implant-associated osteomyelitis.

In vivo MRSA infection in huNSG mice revealed markedly higher CFUs on tibial bone and soft tissue in both huNSG and NSG than C57BL/6J WT mice. Increased MRSA dissemination from the implant to distal organs was also observed in huNSG compared to the control groups confirming their increased susceptibility to *S. aureus*. We found these observations remarkable as several groups have observed no such bacterial dissemination in wild type mouse models of *S*. *aureus* osteomyelitis (56, 57). The increased tibial bacterial load in the bone and MRSA dissemination in humanized mice could be attributed to induction of human immune response due to *S. aureus*, and the presence of staphylococcal immunotoxins that exhibit high tropism to human leukocyte receptors (29, 30). This idea is consistent with the exacerbated lung pathology reported by Prince et al. and the decreased severity in huNSG mice infected with an MRSA strain deficient in the human-specific PVL toxin (42). Another example is the increased susceptibility to MRSA bacteremia in a humanized C57BL/6J mouse containing human CD11b receptor due to strong tropism of immunotoxin LukAB for human CD11b (58). These studies, including ours, highlight the adaptation processes that this pathogen has evolved to survive in the human host.

Analysis of the MRSA-infected tibia in huNSG mice revealed increased bone osteolysis compared to C57BL/6J WT mice. Perhaps the presence of human immune cells in the bone marrow of huNSG, and the ability of *S. aureus* to target human leukocytes are causing increased osteoclastogenesis and infection-associated trabecular bone loss during MRSA osteomyelitis (59). Nonetheless, the increased dysregulation of bone homeostasis during osteomyelitis in huNSG mice warrants further investigation.

An important finding of the current study is the extensive MRSA-induced SAC formation in huNSG mice. Quantitative analyses of the SACs show that the number of SACs per bone area was significantly higher in huNSG mice suggesting increased interaction between *S. aureus* and human leukocytes in the bone. The formation of a multilayered SAC structure during osteomyelitis is a host-induced mechanism of infection control, which is manipulated by *S. aureus* with the deployment of several virulence genes including clumping factor A (ClfA), chemotaxis inhibitory protein of staphylococci (CHIPS), and staphylococcal complement inhibitor (SCIN) (51, 52, 60–62). In clinical studies, the lack of humoral immunity against SCIN and CHIPS correlated with adverse clinical outcomes in patients with *S. aureus* osteomyelitis (49). Assessing the expression of these genes in a huNSG SAC or a 3D *in vitro* model of SAC (63) using bone marrow cells from these animals could shed light on virulence mechanisms associated with increased abscess formation in humanized mice.

T cells are essential for orchestrating anti-*S. aureus* adaptive immunity, and studies have demonstrated their dichotomous roles in protection vs. pathogenesis during infections (64–69). Analysis of human tissue samples in patients with implant-related bacterial biofilm infections indicate the presence of CD4 and CD8 T cells (70, 71), and these T cells were terminally differentiated effector cells (72, 73). However, these observations were not *S. aureus*-specific, and the exact role of T cells in the context of chronic *S aureus* osteomyelitis remains poorly understood. Immunohistopathology of infected huNSG tibia revealed increased numbers of clustered human T cells adjacent to abscesses, suggesting *S. aureus*-mediated human T cell activation and proliferation. Other studies using intraperitoneal infection model in huNSG mice showed increased human T-cell activation and apoptosis due to *S. aureus*, which led to increased bacterial counts and higher mortality rates in mice (40). Conceivably, exacerbated T cell activation could be due to increased expression of T cell targeting superantigens and immunotoxins in huNSG mice.

The current study is limited by inherent deficiencies in the NSG mouse, including limited myeloid lineage development and insufficient functional T cell development (35, 74). Indeed, *S. aureus* infection was more severe in a humanized NSG mice variant that allowed for enhanced human myeloid lineage reconstitution (42, 75, 76). Additionally, we cannot exclude the effects of sublethal γ-irradiation-induced myeloablation in NSG mice before HSC engraftment. This can be examined in NSG mice with engrafted murine bone marrow cells. A pulmonary infection model utilizing NSG mice engrafted with cells of C57BL/6J mice, reported MRSA levels comparable to those in NSG and C57BL/6J control groups (42), ruling the detrimental impact of radiation on the control of the bacterial infection in the lungs. However, radiation could have a different effect on bone immunity. Nevertheless, further studies are warranted to improve this mouse model to make it highly relevant to human musculoskeletal infections, in addition to validating its usefulness to study fracture-related infections, prosthetic joint infections, and evaluating novel experimental immunotherapies.

## Data Availability Statement

The raw data supporting the conclusions of this article will be made available by the authors, without undue reservation.

## Ethics Statement

The animal study was reviewed and approved by Ethical committee of the canton of Grisons in Switzerland.

## Author Contributions

GM: study conception, experimental design, data acquisition and analysis, funding acquisition, and drafting the manuscript. TM, EMS, JD, RR, and SZ: experimental design, data analysis, funding acquisition, and drafting the manuscript. AW, JR-M, MH, KD, KM, MK, and ETS: experimental design, data acquisition, and analysis. All authors contributed to the article and approved the submitted version.

## Funding

This work was supported by AO Trauma Clinical Priority Program Fellowship (GM) with additional support from AO Trauma Clinical Priority Program (EMS, TM), NIH NIAMS P30 AR069655 (EMS), P50 AR072000 (EMS), internal funds from the Department of Medicine in the University of Rochester, and R01AI111914 (JR-M).

## Conflict of Interest

The authors declare that the research was conducted in the absence of any commercial or financial relationships that could be construed as a potential conflict of interest.

## Supplementary Material

Supplementary Figure 1 Flow cytometry gating strategy for analyzing human immune cells in huNSG mice spleen. The sequential gating strategy and representative contour plots for identifying live splenocytes (Live/Dead eF780) and their subpopulations of human T cells (CD3+), T helper cells (CD3+CD4+), cytotoxic T cells (CD3+CD8+), B cells (CD19+), and NK cells (CD56+) their subpopulations of human T, B, and NK cells is depicted here.

Supplementary Figure 2 Serum human cytokine and chemokine expression analyses in humanized mice. Serum samples were collected over time ((PreOP, day 7, day 14 post-op) from huNSG mice infected with either a sterile (Sham) or *S. aureus* contaminated tibial implant. The dotted line indicates the lower limit of detection for each cytokine (n = 20, **p <* 0.05, ***p <* 0.01, two-way ANOVA).

Supplementary Figure 3 μCT analyses of new reactive bone formation. Longitudinal μCT analyses for new reactive bone formation were performed on MRSA-infected or sterile-pin infected huNSG, NSG, and C57BL/6 WT mice to reveal **(A)** Bone Volume, **(B)** Total Volume, and **(C)** Bone Volume Density (BV/TV) (N = 4-6 in each cohort, **p <* 0.05, ***p <* 0.01, ****p <* 0.001, one-way ANOVA).

Supplementary Figure 4 Evidence of human immune cell proliferation in the huNSG tibia due to *S. aureus* implant-associated osteomyelitis. The histology sections described in **Figure 4** were stained with fluorescently labeled antibodies specific for goat anti-proliferating cell nuclear antigen (PCNA), anti-human CD3, and anti-human CD20. Light microscopy of the H&E stained sections **(A,C)**, and fluorescent microscopy of adjacent 5 μm sections **(B,C,E,F)** were performed on the SACs in tibiae from infected huNSG mice, and sham-control huNSG mice. White squares show higher magnification images of the CD3^+^ T cells (red), CD20^+^ B cells (green), and PCNA^+^ cells (white), in the sections of the 3x3 mosaic immunofluorescent micrographs. Note that proliferating PCNA+ human T cell and B cells accumulate around the SACs only in the infected huNSG mice **(E, F)**. Histomorphometry was performed on 5 randomly chosen fields at 200X magnification in each condition **(G-H)**, and aggregated data is presented as the mean+/- SEM for each group (n = 3 mice/group, ***p <* 0.01, ****p <* 0.001, one-way ANOVA). Note that the percentage of proliferating human T cells (CD3+PCNA+ cells) is significantly higher than proliferating B cells (CD20+PCNA+ cells).
