# Supplementary figures and images for: Humanized Mice Exhibit Exacerbated Abscess Formation and Osteolysis During the Establishment of Implant-Associated Staphylococcus aureus Osteomyelitis
Source: Front Immunol. 2021 Mar 18;12:651515. doi: 10.3389/fimmu.2021.651515 (PMC8012494; doi:10.3389/fimmu.2021.651515)

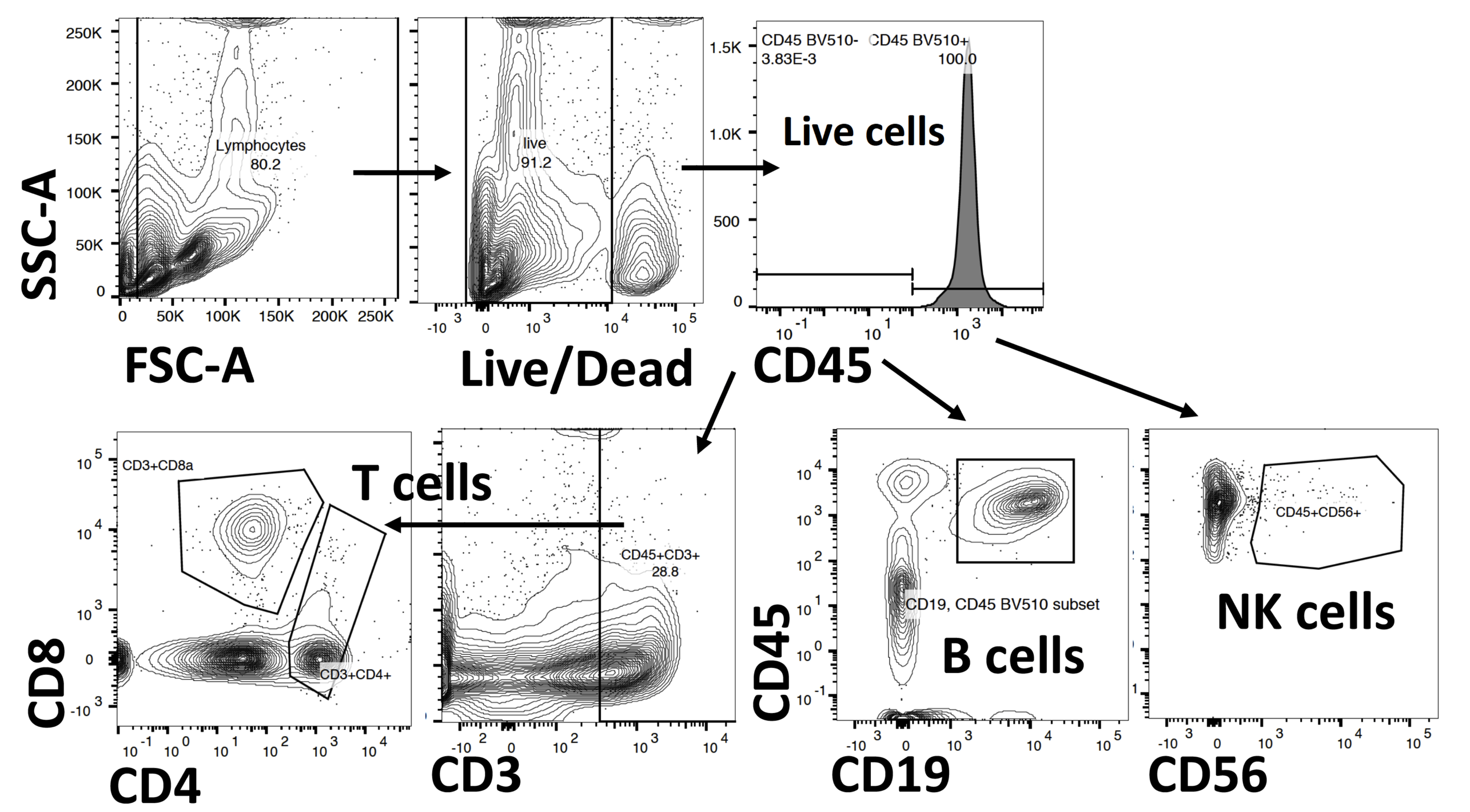

Supplement: Supplementary Figure 1 — Flow cytometry gating strategy for analyzing human immune cells in huNSG mice spleen. The sequential gating strategy and representative contour plots for identifying live splenocytes (Live/Dead eF780) and their subpopulations of human T cells (CD3+), T helper cells (CD3+CD4+), cytotoxic T cells (CD3+CD8+), B cells (CD19+), and NK cells (CD56+) their subpopulations of human T, B, and NK cells is depicted here. [file Image_1.tiff]

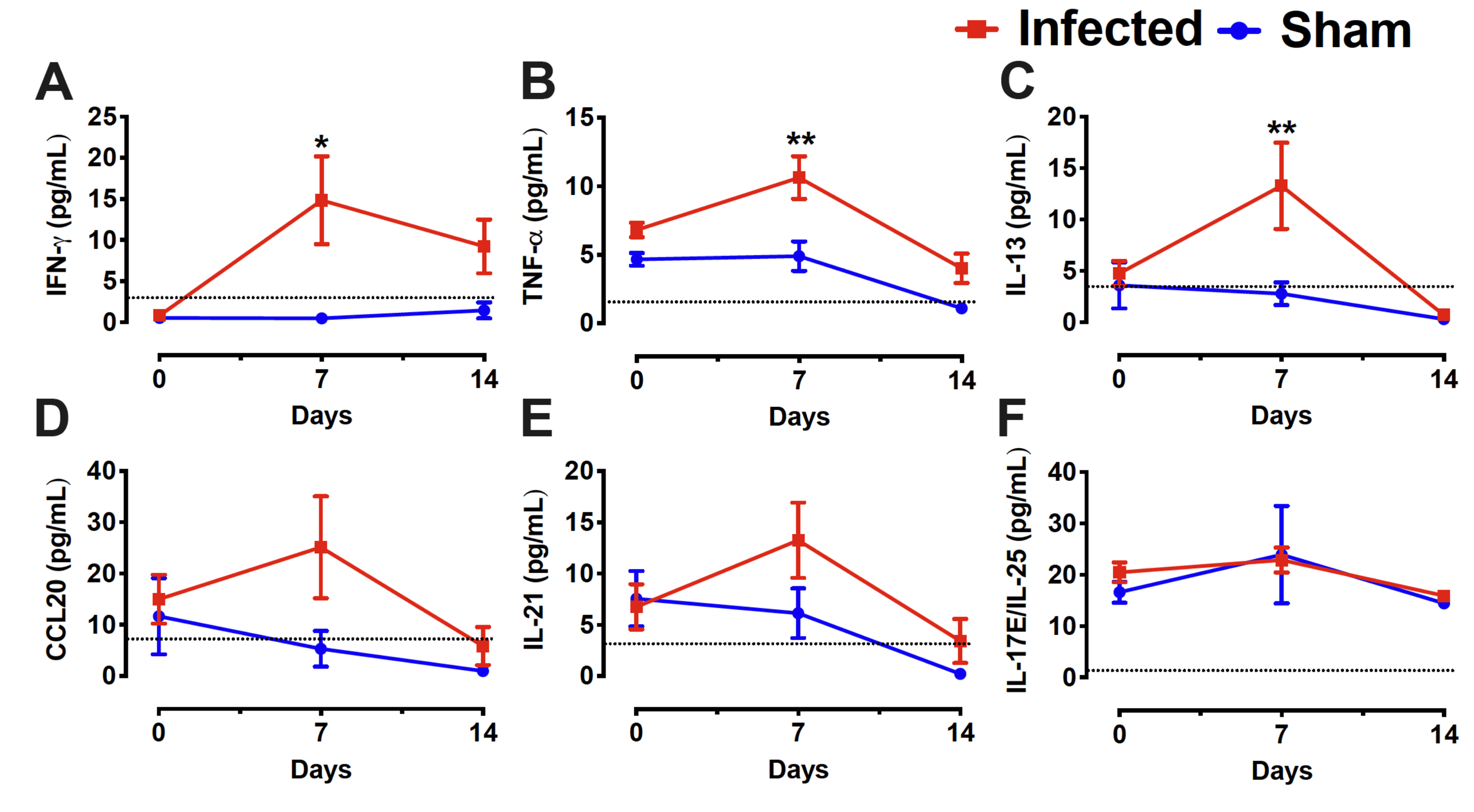

Supplement: Supplementary Figure 2 — Serum human cytokine and chemokine expression analyses in humanized mice. Serum samples were collected over time ((PreOP, day 7, day 14 post-op) from huNSG mice infected with either a sterile (Sham) or S. aureus contaminated tibial implant. The dotted line indicates the lower limit of detection for each cytokine (n = 20, *p < 0.05, **p < 0.01, two-way ANOVA). [file Image_2.tiff]

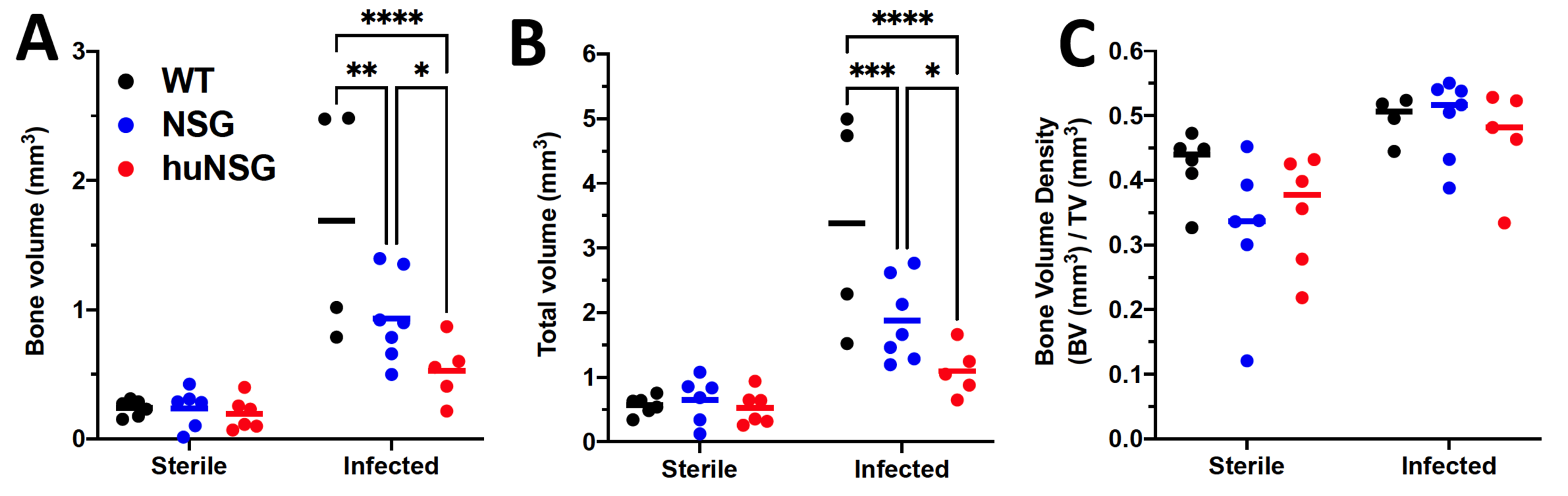

Supplement: Supplementary Figure 3 — μCT analyses of new reactive bone formation. Longitudinal μCT analyses for new reactive bone formation were performed on MRSA-infected or sterile-pin infected huNSG, NSG, and C57BL/6 WT mice to reveal (A) Bone Volume, (B) Total Volume, and (C) Bone Volume Density (BV/TV) (N = 4-6 in each cohort, *p < 0.05, **p < 0.01, ***p < 0.001, one-way ANOVA). [file Image_3.tiff]

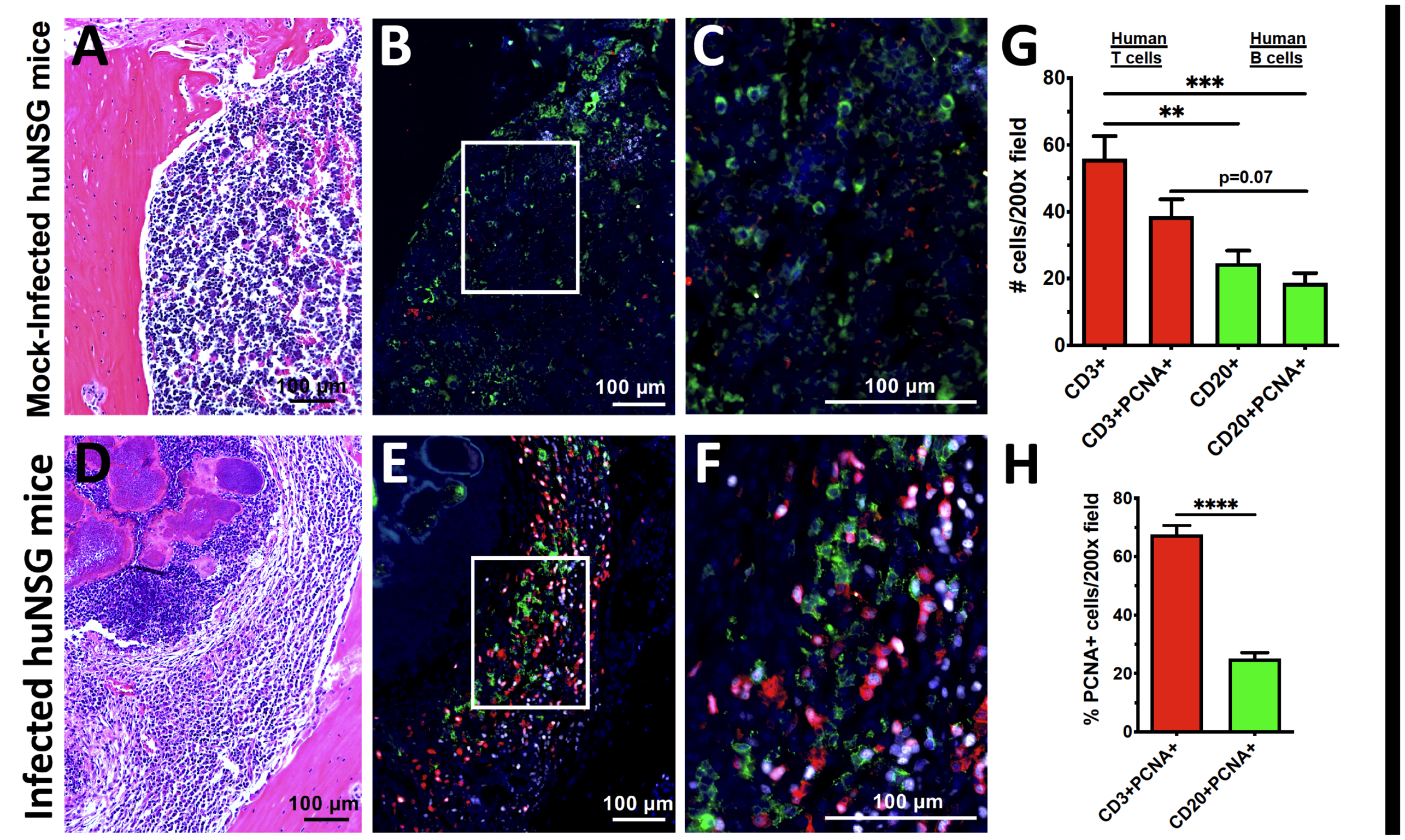

Supplement: Supplementary Figure 4 — Evidence of human immune cell proliferation in the huNSG tibia due to S. aureus implant-associated osteomyelitis. The histology sections described in Figure 4 were stained with fluorescently labeled antibodies specific for goat anti-proliferating cell nuclear antigen (PCNA), anti-human CD3, and anti-human CD20. Light microscopy of the H&E stained sections (A,C), and fluorescent microscopy of adjacent 5 μm sections (B,C,E,F) were performed on the SACs in tibiae from infected huNSG mice, and sham-control huNSG mice. White squares show higher magnification images of the CD3+ T cells (red), CD20+ B cells (green), and PCNA+ cells (white), in the sections of the 3x3 mosaic immunofluorescent micrographs. Note that proliferating PCNA+ human T cell and B cells accumulate around the SACs only in the infected huNSG mice (E, F). Histomorphometry was performed on 5 randomly chosen fields at 200X magnification in each condition (G-H), and aggregated data is presented as the mean+/- SEM for each group (n = 3 mice/group, **p < 0.01, ***p < 0.001, one-way ANOVA). Note that the percentage of proliferating human T cells (CD3+PCNA+ cells) is significantly higher than proliferating B cells (CD20+PCNA+ cells). [file Image_4.tiff]
