# Supplementary material for: Humanized Mice Exhibit Exacerbated Abscess Formation and Osteolysis During the Establishment of Implant-Associated Staphylococcus aureus Osteomyelitis
Source: Front Immunol. 2021 Mar 18;12:651515. doi: 10.3389/fimmu.2021.651515 (PMC8012494; doi:10.3389/fimmu.2021.651515)
Supplement: Supplementary file 5 [file Table_1.docx]

**Supplemental Table S1: Human chimerism analyses from humanized mice**

* Data provided by Jackson Labs at 12 weeks at post engraftment
